# High Quality Maize Centromere 10 Sequence Reveals Evidence of Frequent Recombination Events

**DOI:** 10.3389/fpls.2016.00308

**Published:** 2016-03-23

**Authors:** Thomas K. Wolfgruber, Megan M. Nakashima, Kevin L. Schneider, Anupma Sharma, Zidian Xie, Patrice S. Albert, Ronghui Xu, Paul Bilinski, R. Kelly Dawe, Jeffrey Ross-Ibarra, James A. Birchler, Gernot G. Presting

**Affiliations:** ^1^Department of Molecular Biosciences and Bioengineering, University of Hawaíi at MānoaHonolulu, HI, USA; ^2^Division of Biological Sciences, University of MissouriColumbia, MO, USA; ^3^Department of Plant Sciences, University of California DavisDavis, CA, USA; ^4^Department of Plant Biology, University of GeorgiaAthens, GA, USA

**Keywords:** centromere evolution, DNA damage repair, DNA loss at centromeres, hemicentric inversion, illegitimate recombination

## Abstract

The ancestral centromeres of maize contain long stretches of the tandemly arranged CentC repeat. The abundance of tandem DNA repeats and centromeric retrotransposons (CR) has presented a significant challenge to completely assembling centromeres using traditional sequencing methods. Here, we report a nearly complete assembly of the 1.85 Mb maize centromere 10 from inbred B73 using PacBio technology and BACs from the reference genome project. The error rates estimated from overlapping BAC sequences are 7 × 10^−6^ and 5 × 10^−5^ for mismatches and indels, respectively. The number of gaps in the region covered by the reassembly was reduced from 140 in the reference genome to three. Three expressed genes are located between 92 and 477 kb from the inferred ancestral CentC cluster, which lies within the region of highest centromeric repeat density. The improved assembly increased the count of full-length CR from 5 to 55 and revealed a 22.7 kb segmental duplication that occurred approximately 121,000 years ago. Our analysis provides evidence of frequent recombination events in the form of partial retrotransposons, deletions within retrotransposons, chimeric retrotransposons, segmental duplications including higher order CentC repeats, a deleted CentC monomer, centromere-proximal inversions, and insertion of mitochondrial sequences. Double-strand DNA break (DSB) repair is the most plausible mechanism for these events and may be the major driver of centromere repeat evolution and diversity. In many cases examined here, DSB repair appears to be mediated by microhomology, suggesting that tandem repeats may have evolved to efficiently repair frequent DSBs in centromeres.

## Introduction

Centromeres are required for the faithful segregation of chromosomes during cell division in higher organisms and are usually visible as a primary constriction on the chromosome. The proteinaceous kinetochore that forms atop the centromere interacts directly with the spindle microtubules to affect chromosome movement during cell division. A high incidence of DSB formed during mitosis within and near the centromeres of human and mouse cells carrying mitotic spindle defects provides evidence of spindle-induced centromere shearing (Guerrero et al., 2010). Centromere-proximal DSBs of the kind that can lead to deletion and recombination are well documented and are detectable as paracentric chromosome arm inversions (e.g., tomato Tanksley et al., 1992), centric fusion (Robertsonian) translocations (e.g., human Jacobs, 1981) and nested chromosome fusions (e.g., *Brachypodium* Murat et al., 2010; The International Brachypodium Initiative, 2010), as well as breakdown of sorghum-rice colinearity near centromeres (Bowers et al., 2005).

Centromere-specific retrotransposons (CRs) and long tandem arrays of the 156 nt CentC repeat are key DNA components of maize centromeres (Jiang et al., 2003). Although maize diverged from rice around 50 million years ago, CentC is similar to the rice CentO in length and sequence (Lee et al., 2005), indicating that these repeats have been retained at their respective centromeres for a very long time. Nevertheless, domesticated maize shows reduced CentC levels compared to its wild teosinte relatives (Albert et al., 2010; Hufford et al., 2012; Bilinski et al., 2015).

Centromeric retrotransposons (CR) were first discovered as abundant centromere repeats in sorghum and barley (Miller et al., 1998; Presting et al., 1998). Six CR element families have now been described for maize, and their orthologs in rice and other grasses have been identified (Sharma and Presting, 2008, 2014). CR1, CR2, and CR3 of maize have the ability to target their insertion to centromeres, but little is known about the targeting mechanism, how retrotransposition is regulated, or the role these elements play in centromere function. At least five different CR1 subgroups (R1 through R5) have arisen by genomic recombination (Sharma et al., 2008). CR element-derived tandem repeats (Sharma et al., 2013), and incidents of gene conversion (Shi et al., 2010) and reduced maize-sorghum synteny (Wang and Bennetzen, 2012) in maize centromeres have been reported, but relatively little data is available regarding the frequency of these events. High quality physical maps of one or more maize centromeres will be critical to gaining a clearer picture of centromere evolution, but the lengths of centromeric repeats (7–8 kb for CR elements and tens or hundreds of kb for CentC arrays) cause suboptimal assemblies of centromere regions even in the high quality maize reference genome constructed from inbred B73 (Schnable et al., 2009; Wolfgruber et al., 2009).

We resequenced BACs of the reference genome project (Schnable et al., 2009) that correspond to the active maize centromere 10 region (CEN10), as defined by binding of the centromere-specific histone H3 (cenH3), using PacBio technology. Assembly of these long reads with PacBio's SMRT software allowed closure of nearly all gaps covered by our assembly in CEN10 of the reference genome. The long PacBio reads can span complete CR elements and higher-order repeats (HORs) of CentC, enabling accurate assembly and dating of CR insertions and some CentC HORs.

The improved CEN10 assembly revealed evidence of numerous DSB repaired by homology-mediated intrastrand recombination. By sequencing a CEN10 CentC segment in another inbred we identified a homology-mediated recombination that resulted in the deletion of one CentC monomer and the creation of a new CentC variant. A time frame for the deletion, insertion, and inversion events described, including at least one hemicentric inversion that reshaped CEN10 within the last 16.4 thousand years, is provided by dating CR insertions and segmental duplications.

## Materials and methods

### Preparation and sequencing of BACs

**Figure 1:**
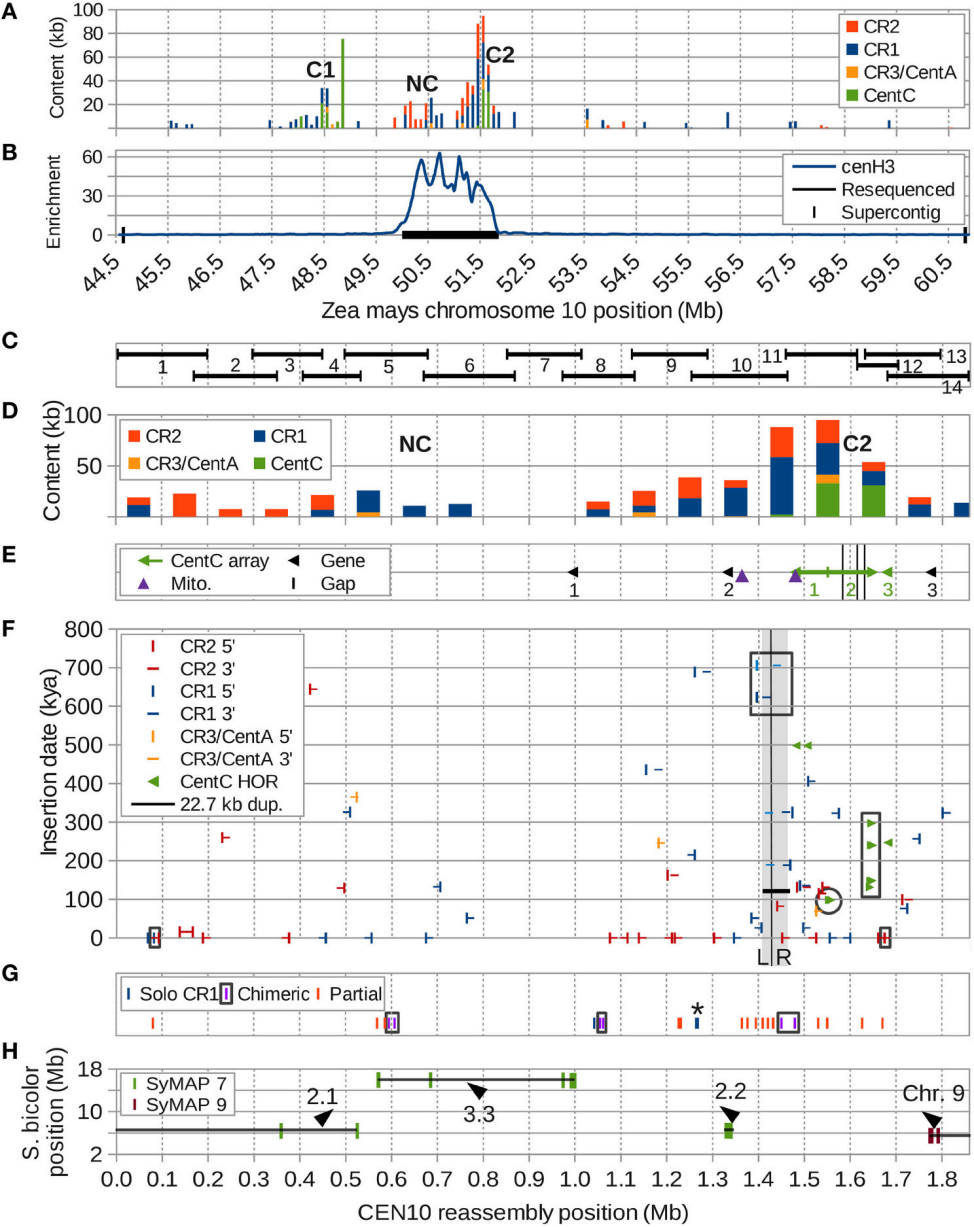
**Overview of CEN10 features. (A)** Two centromeric repeat clusters containing CentC (C1 and C2) in the CEN10-containing supercontig were split by an ancestral hemicentric inversion and are now separated by a 2.6 Mb region including a CR cluster with no CentC (NC). CR3/CentA means CR3 and CentA (nonautonomous CR3). **(B)** Enrichment of anti-cenH3 ChIP-seq reveals NC and C2 are in the active centromere. The resequenced region is indicated by the black horizontal bar. **(C)** The regions of the resequenced CEN10 spanned by each BAC are indicated. **(D)** Centromeric repeat content per 100 kb window. **(E)** Positions of the three CentC arrays (#1 and #2 are interrupted by retrotransposons), three transcribed genes (probed by FISH), two mitochondrial insertions and three remaining sequence gaps (black vertical bars within CentC array #2). Arrows indicate the orientation of the CentC arrays; the junction of two adjoining inverted arrays is marked by a vertical bar. Arrowheads indicate direction of transcription for the three genes. **(F)** Estimated dates of CR element insertion, duplication of the 22.7 kb region (horizontal black line, the duplication is marked by adjacent gray segments labeled “L” and “R” and separated by a vertical black line) and seven higher-order repeats (HORs) of CentC (paired, mostly overlapping, green arrows) are indicated on the y axis. Three CRs with internal recombinations (including a CR partially in the 22.7 kb duplication) are boxed (black). The youngest of the dated CentC HORs (**Figure 5**) is circled, HORs from **Figure 6** are boxed. **(G)** Positions of recombinant CR sequences, including three solo CR1 LTRs (^*^ = two solos separated by only 2.3 kb), three chimeric elements (complete but with mismatched TSDs, LTR pairs are boxed) and 14 partial CR elements. For clarity, only the two closest ends of a chimeric element located in the 22.7 kb duplication are shown. A partial CR1 that may be artificially truncated by BAC (#12) vector is shown at position 1.63 Mb. **(H)** Synteny markers shared with sorghum chromosomes 7 (SyMAP 7) and 9 (SyMAP 9) are indicated by their sorghum and maize coordinates with orientation of blocks (lines) indicated by arrowheads. The most downstream marker cluster of the second synteny block (at 1 Mb) is inverted relative to sorghum, but co-linear with *Brachypodium* and rice. The 1.85 Mb CEN10 is composed of four different syntenic segments from two different sorghum chromosomes indicated here with labels as described in **Figure 7** and Supplemental Figure S10.

Thirteen BACs from the CEN10 region (Schnable et al., 2009; Wei et al., 2009; Wolfgruber et al., 2009) were resequenced using long read PacBio technology. One additional BAC missing from the reference genome (#11 in Table S1 and Figure 1) was identified in GenBank based on its end sequences (accessions ED551002.1 and ED551003.1) matching the flanking BACs and included in our assembly to close a supercontig break in the maize physical map (Soderlund et al., 2000; Wei et al., 2009). BACs were sequenced using both PacBio single molecule sequencing (Eid et al., 2009) and Illumina paired-end sequencing (Bentley et al., 2008). PacBio and Illumina data are available from NCBI under SRA study accession SRP068233.

For DNA preparation, cells carrying BACs were grown in LB medium supplemented with 12.5 μg/ml chloramphenicol. BAC DNA was isolated using the QIAGEN Large-Construct Kit (QIAGEN Sciences, Inc., Germantown, Maryland, USA). For PacBio sequencing 6–16 μg of BAC DNA was isolated. Samples were sequenced using XL-C2 chemistry and MagBead loading with a PacBio RS II sequencer (Pacific Biosciences of California, Inc., Menlo Park, California, USA) at the University of California Davis Genome Center (Davis, California, USA). SMRT cell versions 1.3.1 through 2.0.0 were used. For Illumina library preparation 5 μg of BAC DNA was fragmented using NEBNext dsDNA Fragmentase enzyme (New England Biolabs, Ipswich, Massachusetts, USA) to obtain fragments with maximum size of 300–400 bp. Fragments in the 200–300 bp size range were gel purified using QIAGEN Gel purification kit followed by end-repair of DNA using NEBNext End Repair Module and dA-tailing using NEBNext dA-Tailing. DNA clean up following end-repair and dA-tailing was done using Agencourt AMPure XP reagent (Beckman Coulter, Inc., Brea, California, USA). To each BAC, equimolar stocks of a universal adapter and unique index adapter was ligated using Enzymatics ligase (Enzymatics, Inc., Beverly, Massachusetts, USA) following standard protocols and then the reaction was treated with proteinase K. The ligation reaction was run on an agarose gel and fragments sized 400–500 bp were purified using QIAGEN Gel purification kit. A single cycle of PCR was run with Illumina forward and reverse primers and PCR cleanup was done using AMPure beads. The library quantification was done using qPCR standards from Kapa Biosystems (Wilmington, Massachusetts, USA) and integrity of samples was determined using Bioanalyzer (Agilent Technologies, Inc., Santa Clara, California, USA). Illumina MiSeq sequencing (Illumina, Inc., San Diego, California, USA) was done at the Evolutionary Genetics Core Facility at the Hawai‘i Institute of Marine Biology (Kaneohe, Hawai‘i, USA).

### Assembly of PacBio reads and quality control

BACs were assembled by loading the PacBio SMRT cell data into SMRT Analysis software version 2.3.0 (https://github.com/PacificBiosciences/SMRT-Analysis) and using the HGAP2 protocol (Chin et al., 2013). Custom settings for minimum subread length, minimum seed read length, and estimated insert (“genome”) size for each BAC are described in Table S1. The resulting HGAP2 assembly was then run through the SMRT Analysis Resequencing protocol using unambiguously mapped reads of any size to generate a final insert consensus.

Overlaps between BAC inserts were identified using BLAST (Camacho et al., 2009) and removed. Gaps were inserted into a CentC array of BAC #11 not spanned by a single PacBio read (position 1,580,181–1,580,281 nt), between the non-overlapping BACs #11 and #12 (position 1,610,075–1,610,175 nt) and within a CentC cluster in BAC #12 at the break in identity with BAC#13 (position 1,617,643–1,627,252 nt) caused by a deletion in the resequenced BAC #12. The CEN10 assembly (Data sheet S1 and GenBank accession KT989678) was then integrated into the reference genome (B73 RefGen_v3 via ftp://ftp.ensemblgenomes.org/pub/plants/release-18/fasta/zea_mays/dna/Zea_mays.AGPv3.18.dna.*.gz; Schnable et al., 2009) by replacing the original RefGen_v3 chromosome 10 region from 50,003,470–51,845,973 nt with the 1,852,772 nt reassembly. The regions of AC209849.4 (BAC #12) missing from the resequenced BAC #12 were identified by BLAST2seq, verified visually using JunctionViewer (JV) images and extracted into a separate FASTA file (Data sheet S2).

Cinful retrotransposon sequences in BACs #11–13 were scrutinized in detail using Sanger sequence from GenBank (where available) and Illumina and PacBio data from the resequencing effort, to verify SNPs that suggested a segmental duplication. Illumina read pairs (parameters “-X 600 --no-mixed --no-discordant --no-dovetail”) were mapped to the PacBio assemblies of each BAC using Bowtie 2 (Langmead and Salzberg, 2012), and the consensus was obtained using the Integrative Genomics Viewer (Robinson et al., 2011). Consensus sequences from Sanger reads (available for BACs #12–13 from the maize reference genome project) were generated using the Geneious software program (Kearse et al., 2012) to map reads to each PacBio assembly reference sequence (medium sensitivity). Cinful elements were extracted from each consensus and aligned using MUSCLE (Edgar, 2004).

The CR and CentC content was calculated for each assembled BAC insert and the Illumina reads generated from each BAC using RepeatMasker with rmblast (repeatmasker.org) and the consensus sequences also used for JV annotations (Wolfgruber and Presting, 2010).

Fluorescence *in situ* hybridization (FISH) was conducted as previously described (Kato et al., 2006; Lamb et al., 2007a) using sequences from the CEN10 assembly. Gene probes were generated by PCR amplification of B73 genomic DNA using primers that amplify fragments corresponding to positions 991,393–999,754 nt (gene 1), 1,331,762–1,340,808 nt (gene 2), and 1,771,713–1,780,104 nt (gene 3).

### Dating retrotransposon insertions and genome duplications

LTR retrotransposons that inserted within the CEN10-containing supercontig (original reference genome (v3) positions 44,645,284–60,809,161 nt) were dated. RepeatMasker with rmblast was used to identify CR LTRs using the CR1, CR2, CR3, and CentA LTR sequences used for JV annotations and non-CR LTRs using sequences annotated as “LTR” from the maize subset of the GIRI Repbase (Jurka et al., 2005). Non-CR LTRs were identified only from sequence not already identified as CR LTR. Dates were calculated for pairs of LTRs with identical 5 nt flanking target site duplications (TSDs) and only when at least 2 nt on the edges of LTRs had reverse complement matches, e.g., 5′-TG with CA-3′. Additionally, LTRs had to belong to the same subfamily, e.g., CR1, and orientation, and the smaller LTR could be no less than 90% of the longer LTR length. CR elements were also dated using JV annotations as a guide (Figure S1), allowing for single mismatch/indel between TSDs.

Insertion times were estimated by aligning LTR pairs using MUSCLE (Edgar, 2004) and calculating a Kimura 2-parameter (K2p) value (Kimura, 1980) from the resulting alignment using BioPerl (http://www.bioperl.org/wiki/Main_Page). The K2p value was translated into years using the previously determined substitution rate of 3.3 × 10^−8^ substitutions/site/year (Clark et al., 2005).

### Generation and mapping of anti-cenH3 ChIP-Seq reads

Polyclonal rabbit antibody was generated (Cocalico Biologicals Inc., Reamstown, PA, USA) from the purified 58 N-terminal amino acids (1–58) of maize cenH3 protein produced in *Escherichia coli* after cloning into pET19 with N-terminal 6xHis tag. The protein was purified with a nickel column following standard protocol. The antibody serum was affinity-purified with antigen-coupled column prior to use.

Immature ears (about 7 cm long) from the maize cultivar B73 inbred line were used for chromatin immunoprecipitation (ChIP). ChIP experiments were performed according to previously published protocols with some modifications. In brief, plant material was ground to a fine powder in liquid nitrogen using mortar and pestle. The powder was cross-linked with 1% formaldehyde in cross-linking buffer (0.4 M sucrose; 10 mM Tris-HCl, pH 8.0; 1 mM EDTA; 1 mM PMSF) for 20 min on ice, and cross-linking was stopped by adding 0.1 M glycine (final concentration) for another 5 min on ice. After filtering through two layers of miracloth, the crude nuclei were isolated using M1 (11.9% hexylene glycol; 10 mM KPO4, pH 7.0; 100 mM NaCl; 5 mM beta-mercaptoethanol; 0.1 mM PMSF, plant protease inhibitor cocktail) and M2 buffer (8.85% hexylene glycol; 10 mM KPO4, pH 7.0; 10 mM MgCl2; 0.5% Triton X-100; 5 mM beta-mercaptoethanol; 100 mM NaCl). Chromatin in the crude nuclei preparation was digested with Micrococcal Nuclease (MNase) in MNB buffer (50 mM Tris-Cl, pH 8.0; 1 mM CaCl2; 4 mM MgCl2; 0.3 M sucrose) at 37°C to produce mono- and oligo-nucleosomes. After clearing with protein A dynabeads (Invitrogen / Thermo Fisher Scientific, Waltham, MA, USA; Cat. no. 100-02D), the chromatin was incubated with purified anti-(*Zea mays*) cenH3 antibody. Rabbit IgG antibody was included as a negative control. After overnight incubation by rotating in a 1°C cold room, the antibody-chromatin complex was immuno-precipitated with protein A dynabeads, followed by washing, elution, reverse cross-link and DNA purification. After ChIP quality was confirmed by qPCR, 10 ng of ChIPed DNA were used for 101 cycle paired-end Illumina sequencing (University of Utah, Salt Lake City, Utah, USA). Input (chromatin) DNA was cut from an agarose gel and sequenced. CenH3 ChIP-seq data for inbred B73, as well as the mononucleosome fraction of MNase-digested input DNA was deposited to NCBI under SRA study accession SRP067358.

Input and anti-cenH3 ChIP-seq Illumina read pairs were mapped to RefGen_v3 with the revised CEN10 (including all reference chromosome, mitochondrial, and plastid DNA sequences) using Bowtie 2 (Langmead and Salzberg, 2012) (parameters “-X 1000 --no-mixed --no-discordant --no-dovetail”). Both reads had to match exactly to the reference and at least one had to map uniquely in the genome. Enrichment and coverage by input or ChIP-seq reads were determined using samtools (Li et al., 2009). Nucleotide coverage was summed for each 100 kb window overlapping by 10 kb. An average was then calculated over 9 windows and normalized to the number of read pairs in the corresponding dataset. Enrichment was calculated in 10 kb increments across the genome by dividing ChIP-seq over input.

### Generation of a JV image spanning CEN10

A JV image of reassembled CEN10 was generated (Figure S1). JV annotations: CR2 LTR red, CR1 LTR blue, CR CDS tan, CR3 LTR pink, CentA LTR orange, CentC green, non-CR repeat or CR2 UTR gray, mitochondrial DNA purple, and expressed rice genic sequence yellow. Annotations obtained via cross_match (boxes) are drawn above the BLAST results (orientations indicated by arrows). All dated elements or solo LTRs were labeled above their LTR annotations (boxed numbers), with positive and negative numbers indicating insertion date in thousands of years and solo LTRs, respectively. Sequences at the ends of LTRs are shown. MUMmer (Kurtz et al., 2004) minimum match lengths ≥ 20 nt are shown at the bottom of the image, drawn longest match to shorter matches limited by what would fit in the space.

### Analysis of recombined CR sequences

A table describing CR sequences in CEN10 was generated for each CR sequence having overlapping cross_match and BLAST CR homology annotations in the JV image of CEN10 (Figure S1). CR sequences with multiple BLAST HSPs in their coding sequences (CDSs) were investigated for recombination at the nucleotide level. These potential recombinants were extracted and aligned to all uninterrupted/complete elements (no CR or other sequence insertions, and no deletions) of the same type (CR1 or CR2) in CEN10 using MUSCLE. The resulting sequence alignments were visualized using UGENE (Okonechnikov et al., 2012) to determine deletion and insertion characteristics.

### Characterization of CentC HORs

Full-length CentC monomers were identified in high-confidence reassembled sequences between positions 1,476,916–1,564,121 nt and CentC array #3 of the reassembly. Full-length monomers were identified as aligning to ≥147 nt of a consensus CentC (Data sheet S3) using BLASTN 2.0 (WU-BLAST; http://blast.wustl.edu/). These monomers were numbered in 5′ to 3′ order relative to the CEN10 assembly. A multiple sequence alignment of the monomers was constructed by MUSCLE, and a bootstrapped (1,000) neighbor-joining tree was generated using MEGA (Tamura et al., 2013). CentC HORs were identified using the tree. The three longest HORs (contained in arrays #1–2 in Figure 1), as well as the longest HOR in the smallest CentC array (array #3 in Figure 1) were dated by joining the internal full-length CentC monomers from each HOR, aligning them using MUSCLE, and generating a date from the alignment (see Dating Retrotransposon Insertions and Genome Duplications).

### Cloning and analysis of a CentC fragment in cultivars B73 and Mo17

Equivalent CentC fragments from the CEN10 of maize inbreds B73 and Mo17 were cloned into the highly stable pJAZZ (Godiska et al., 2010) linear vector. The Mo17 fragment was cloned directly from whole genome DNA without PCR amplification (Data sheet S4 and GenBank accession KT989679) and the B73 fragment was subcloned using the BAC (#13 ZMMBBb-410L22 in Table S1) in the CEN10 tiling path. Young maize cultivar Mo17 tissue was ground with liquid nitrogen and incubated in a DNA extraction buffer before DNA was extracted using chloroform, then precipitated using ethanol. The precipitant was subsequently digested overnight at 37°C with *Hae*III, precipitated again, and end-repaired using the Lucigen DNATerminator End Repair Kit (Lucigen Corp., Middleton, WI, USA). End-repaired DNA in solution was run through a 0.6% agarose gel and visualized via SYBR Safe DNA Gel Stain (Life Technologies, Grand Island, NY, USA) under blue light. Bands ≥8 kb to the gel wells were cut out and purified using the Epoch gel extraction kit (Epoch Life Science, Missouri City, TX, USA). Ethanol precipitated DNA from gel extract was then ligated into pJAZZ vectors using BigEasy v2.0 Linear Cloning Kit (Lucigen Corp.) and transformed into *E. coli* using the procedure outlined in the BigEasy Kit. Incubated transformants were plated onto kanamycin YT-Agar with X-gal and IPTG. White colonies were picked into water and restreaked, then PCR screened using vector primers (SL1 5'-CAGTCCAGTTACGCTGGAGTC-3' and NZRevC 5′-AAATGGTCAGTTAATCAGTTCT-3′) and CentC primers (CentC_F 5′-TCCAAAACTCATGTTTGGG-3′ and CentC_R 5′-GTGGATTGGGGCATGTTCG-3′). PCR products were run through a 2% agarose gel to identify monomer/dimer/trimer bands of the 156 nt CentC repeat. Clones with the expected band sizes were grown in TB medium with kanamycin and arabinose induction solution overnight at 250 RPM and 37°C. Plasmids were then isolated using the QIAGEN Miniprep kit (QIAGEN Inc., Valencia, CA, USA). The vector-containing solution was treated with *Not*I at 37°C to release the insert and run on a 0.6% agarose gel. A clone with a lane containing three strong bands for two vector arms and one insert was identified, and the insert band was cut and purified using the MACHEREY-NAGEL NucleoSpin Gel and PCR Clean-up kit (Bethlehem, PA, USA). Purified DNA was sonicated for 20 s, end-repaired, run on a 1% agarose gel, and bands 1–8 kb extracted then transformed into pJAZZ vector by electroporation. Colonies were then screened for CentC monomer/dimer/trimer bands using PCR and a 2% agarose gel as previously described, then DNA from CentC-containing colonies were Sanger sequenced at Pacific Biosciences Research Center Biotech Core (Honolulu, HI, USA) using vector primers SL1 and NZRevC. Sanger sequences (available at NCBI Trace Archive TI numbers 2343263554-2343263871) were assembled in Consed (Gordon et al., 1998) where discrepancies between assembled reads were manually edited before generating a consensus sequence. The B73 BAC DNA was isolated using the QIAGEN Large-Construct kit (QIAGEN Inc.) then subcloned into pJAZZ, screened, sequenced, and assembled as done for the Mo17 DNA.

A divergence date between the B73 and Mo17 CentC sequences was calculated by generating a MUSCLE alignment of their CentC segments, calculating a K2p distance from the alignment, then calculating a date from the K2p distance as was done to date CR insertions.

Full-length CentC monomers were identified in the B73 array, HORs were identified from a phlogenetic tree of the monomers, and full-length monomers were concatenated and dated as previously described. The HORs were additionally redefined using the MUMmer (--maxmatch) annotations in JV by moving the HOR borders according to a longest sequence match.

### Mapping of sorghum syntenic markers

Conserved single-copy sequences in pericentromeres (CSCP) markers (Wang and Bennetzen, 2012) are expected to flank ancestral centromeres and were mapped to the revised reference genome using MUMmer (end-to-end unique mapping). Independently, genic sorghum-maize synteny markers were identified with the SyMAP software (Soderlund et al., 2011) run on a local computer and comparing the revised maize reference genome against only those sequences of the sorghum early release version 2.1 reference genome that correspond to annotated genes (Paterson et al., 2009). Sorghum sequence and gene positions were obtained from Phytozome (http://www.phytozome.net/). Syntenic markers were grouped into blocks manually after visualizing the data.

### Determining expression of genic sequences in CEN10

Gene sequences in CEN10 were labeled according to RefGen_v3 annotations at MaizeGDB.org (Andorf et al., 2015). Gene annotated sequences in the JV image of reassembled CEN10 (Figure S1) were translated to original RefGen_v3 positions using (gene overlapping) SyMAP markers. Expression was determined by mapping maize cultivar B73 RNA-seq data (NCBI BioProject accession PRJNA219741) to the reassembled CEN10 sequence using TopHat (Trapnell et al., 2009).

### Identification of CR and CentC content in the CEN10 supercontig

To identify CR and CentC content across the supercontig containing CEN10 a competitive BLAST was performed as previously described (Schnable et al., 2009) except that 1) CentC was included with the CRs and 2) BLAST was used instead of WU-BLAST.

## Results

### Resequencing dramatically improves assembly of CEN10

The centromere region of maize chromosome 10 contains three clusters of centromeric repeats (Figure 1A), C1 and C2, which contain tandem CentC clusters, and NC, a CR-rich region devoid of CentC. CentC regions of C1 and C2 are separated by 2.6 Mb, can be visualized as two distinct FISH signals (e.g., https://birchler.biology.missouri.edu/wp-content/uploads/2015/06/166-33_B73.jpg) and likely are the result of a hemicentric inversion (Lamb et al., 2007b). The active centromere 10 (CEN10) of inbred B73, i.e., the region covered by cenH3 nucleosomes (Figure 1B), includes NC and C2, and represents a neocentromere colonized by cenH3 nucleosomes a few thousand years ago (Schneider et al., 2016).

Fourteen overlapping BACs spanning the active CEN10 (Figure 1C), including one selected to close a known gap between supercontigs, were sequenced to high depth using PacBio (Table S1) and Illumina (Table S2) technologies. The percentage of CentC and CR content of the PacBio BAC assemblies differed from that of the Illumina reads by only 0.1–2.8% (Table S2) of the BAC insert size, indicating that the assemblies accurately reflect the repeat content of each BAC. However, our assembled BAC #12 insert is much smaller (63,205 nt) than the corresponding GenBank sequence AC209849 (164,526 nt), due to loss of a substantial portion of the BAC insert (including 75,222 nt CentC and 6,865 nt of CR) via CentC-mediated recombination during propagation (Data sheet S2).

BAC #11, which was originally selected to close a supercontig break (Wei et al., 2009), ends in a Cinful element. High SNP rates between that Cinful element compared to that shared by BACs #12 and #13 (6 SNPs in 4,408 nt, Table S3) relative to the calculated sequencing error rate (1 SNP per >147 kb see below) suggests that these elements are the result of a recent segmental duplication. Independently generated Illumina and, where available, Sanger sequence data confirmed the 6 SNPs detected in the PacBio sequence and thus the presence of a segmental duplication in this region. Therefore, a supercontig break remains in our assembly (position 1,610,075–1,610,175 nt) and BAC #11 does not overlap with BAC #12.

Independently assembled sequence from overlapping BAC inserts (Figure 1C) revealed error rates of 1 SNP per 147,437 nt and 1 indel per 18,430 nt (Table S4). Most indels (22/24) involved a single nucleotide at the ends of mononucleotide runs ranging from 5 to 33 nucleotides in length (10.2 ± 5.8 nt). All BAC overlaps were merged to produce a 1.85 Mb CEN10 sequence (Data sheet S1) that contains a total of 237,593 nt CR1, 177,586 nt CR2, 17,630 nt CR3 and CentA, and 58,169 nt CentC. The resequenced CEN10 contains only three gaps, one of which is located in a large CentC cluster of BAC #11 that could not be spanned by PacBio reads, another represents a gap in the minimum tiling path between BACs #11 and #12 and the third is due to sequence lost in our BAC #12.

The number of correctly assembled CR elements with matching LTRs increased from five in the reference genome to 42 (prior to including sequences contributed by BAC #11). The BAC #11 sequence completes two CRs in BAC #10 and adds 11 additional CR elements with matching LTRs (total of 13). In this assembly we corrected five CRs with mismatched TSDs and five solo LTRs that had been improperly assembled in the reference genome.

### Distribution of centromeric repeats in CEN10

Two CR-rich clusters within CEN10 are separated by 272 kb (Figure 1D). The downstream cluster contains the three CentC arrays of C2 (Figure 1E), the first two of which point in opposite directions and contain numerous CR insertions, and a third small (< 5 kb) CentC array that lacks retrotransposon insertions. The insertion times of the CR elements in the two CR-rich clusters range from 0 to 650 kya (Figure 1F), but the downstream CR cluster contains a much larger number of solo, chimeric, and partial CR elements than the upstream NC cluster (Figure 1G). Syntenic markers strongly suggest that NC and C2, now separated by a non-syntenic region 3.3 used to be adjacent to each other (Figure 1H). Taken together the data in Figure 1 suggest that C2 marks the ancestral centromere location, before one or more hemicentric inversions moved some of the old CR elements to NC and inserted the previously non-centromeric region 3.3 into CEN10.

### Genes in CEN10

Three genes that lie very close to the ancestral centromere containing the CentC cluster have been confirmed by FISH (Figure 1E and Figure S2). Gene 1 (GRMZM2G137715, expressed in leaves) is located 329 kb upstream of gene 2 (GRMZM2G361718, expressed in root), which lies immediately adjacent to a recently inserted CR1 and just upstream of the most centromere repeat-dense region of CEN10. A third gene, created by merging GRMZM2G101098 with GRMZM5G846522 based on RNA-seq data (not shown) after correctly assembling this region, lies < 100 kb downstream of the final CentC cluster and is highly expressed in young tissues (MaizeGDB.org).

### Evidence for frequent DSBs in CEN10

Evidence for numerous DSBs detected in the CEN10 reassembly in the form of recombined or deleted repeat sequences, and genomic rearrangements relative to the sorghum genome, are listed below. Where possible, deletions within CR elements were dated based on insertion time of the retrotransposon, and duplications were dated based on divergence of the original and duplicated sequences.

#### Lost and recombined CR sequences

**Figure 2:**
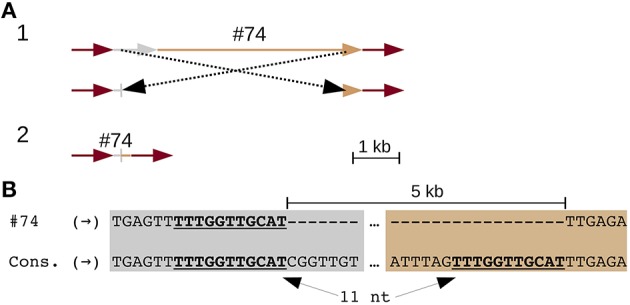
**An internal CR deletion by homology-mediated recombination**. CR2 element (#74 in Table S5) has identical LTRs, which date the insertion and subsequent recombination of this element to < 16.4 kya. **(A)** Line drawing illustrates (part 1) recombination between UTR (gray) and CDS (tan) leading to the observed truncated CR identified in CEN10 (part 2). The CR2 LTRs are colored red. **(B)** Alignment of the UTR and CDS deletion junction to consensus CR2 sequences illustrates that the deletion is mediated by 11 nt of identical sequence (underlined).

Three solo LTRs (Figure 1G), seven CR elements with large internal deletions (two of which share the same deletion) (Figure 2 and Figure S3), and three chimeric CR elements (flanked by mismatched TSDs) reveal frequent recombinations in CEN10. CEN10 contains 14 partial CR sequences (e.g., a single LTR joined to incomplete CDS), excluding segmentally duplicated partials, solo LTRs, and an element truncated by BAC vector (Figure 1G and #70 in Table S5). Partial elements likely result from homology-mediated intrastrand recombination, as seen in the recently inserted CR2 (identical LTRs indicate insertion time < 16.4 kya) that has a 5 kb internal deletion bordered by 11 nt of identical sequence (Figure 2). Other internal CR deletions have 1–3 identical nucleotides flanking the deletions (Figure S3).

**Figure 3:**
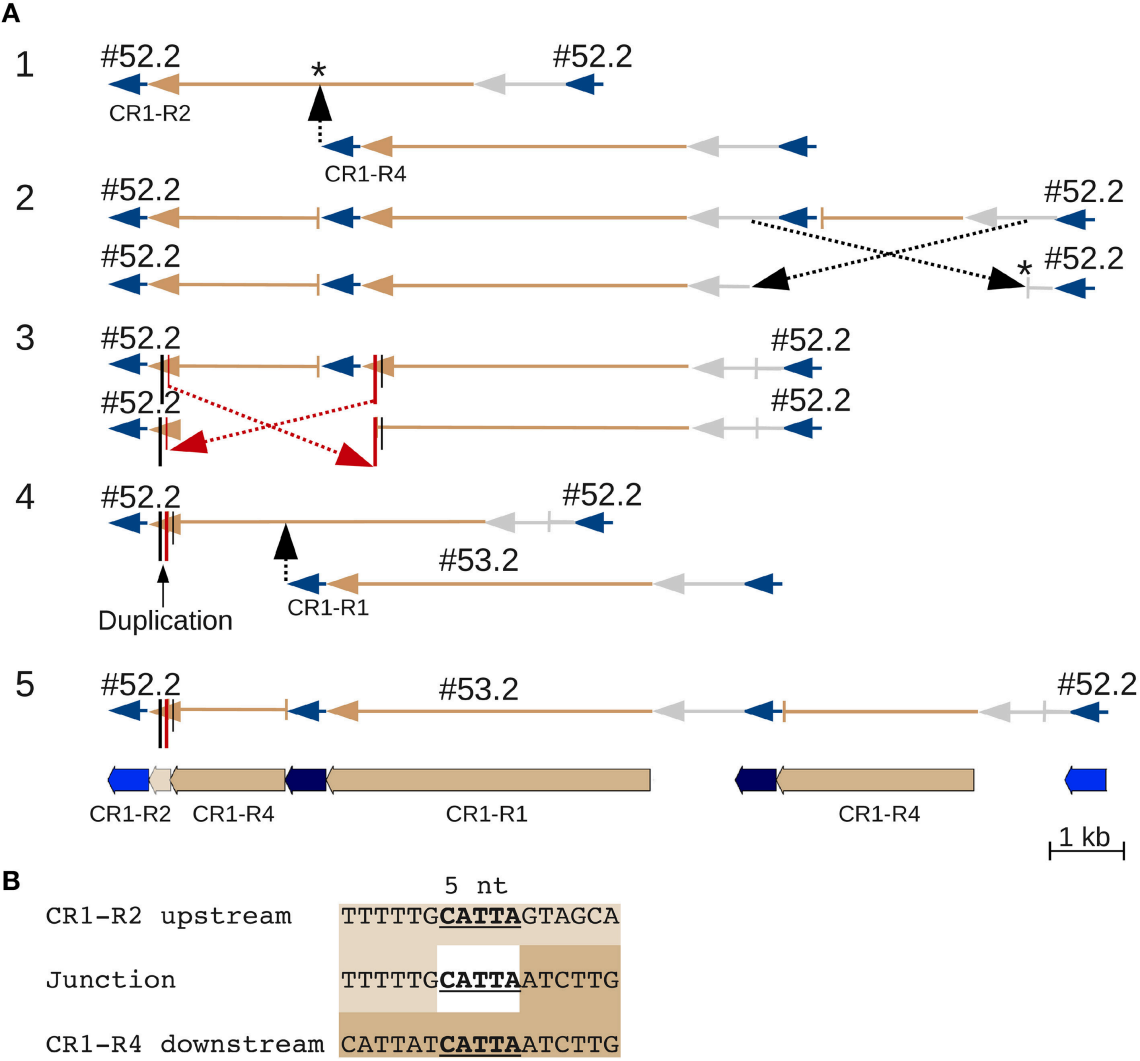
**A CR CDS duplication from nested insertion and recombination**. Two CR1 elements that at first glance appear to represent a simple nested insertion, with insertion times of 326 kya and 188 kya for #52.2 and #53.2, respectively (Table S5). Closer examination reveals that element #53.2 (belonging to one of the five (R1 through R5) CR1 subgroups (Sharma et al., 2008)) inserted into a chimeric CR1 element that contains an apparent ~162 nt duplication. This chimeric element is the result of an intrastrand recombination between two different 5 nt regions of the R4 and R2 element, specifically between the downstream CR1-R4 and the upstream CR1-R2 region that adds 162 nt to the polyprotein region of this element. **(A)** Line drawings showing the events leading to the current sequence: original insertion of a CR1-R4 into a CR1-R2 (^*^ indicates that the precise insertion site is unknown) was followed by two recombinations resulting in loss of the R4 LTRs. Because the second recombination involved two offset positions (red in part 3), it appears like a duplication that can only be identified as a recombination product because the upstream, R4-derived, and the downstream, R2-derived, copy contain different alleles at the ends of their CDS. JV annotations are shown in part 5 (CR1 LTRs blue, CDS tan, UTRs not annotated). **(B)** Alignment showing 5 nt of identical sequence between the alternative-end joining junction and the corresponding regions in R2 and R4. Line drawings colored like JV annotations and UTRs in gray.

CR1 #52.2 contains what appears to be a 162 nt duplication in its polyprotein coding sequence that really is due to double recombination between nested CR1 elements (Figure 3). One recombination involving five nucleotides of identical sequence that are repeated in the CR1 polyprotein joined the downstream region of one element with the upstream region of the second element, and could only be distinguished from a local duplication because the two elements belong to different subtypes. In another example, two CR1s inserted into, and recombined with, a third CR1 (Figure S3C), creating two chimeric elements.

#### Partial mitochondrial sequences

Two fragmented mitochondrial sequence clusters in CEN10 (Figure 1E) provide further evidence of double-strand DNA breakage in the centromere. The clusters are located 115 kb apart and align to two and three different regions of the mitochondrial genome, respectively. The order and orientation of these nuclear mitochondrial fragments relative to the maize mitochondrial genome suggest that homology-mediated recombination reduced an initially much larger mitochondrial insert into these fragments (Figure S4). In fact, examination of the junctions resulting from these deletions relative to the maize mitochondrial reference sequence confirmed that two of the deletions involved homologies of at least three identical nucleotides. The third deletion may have occurred via multiple events or represent a region where the mitochondrial reference genome differs from the inserted sequence.

#### A large adjacent segmental duplication in CEN10

**Figure 4:**
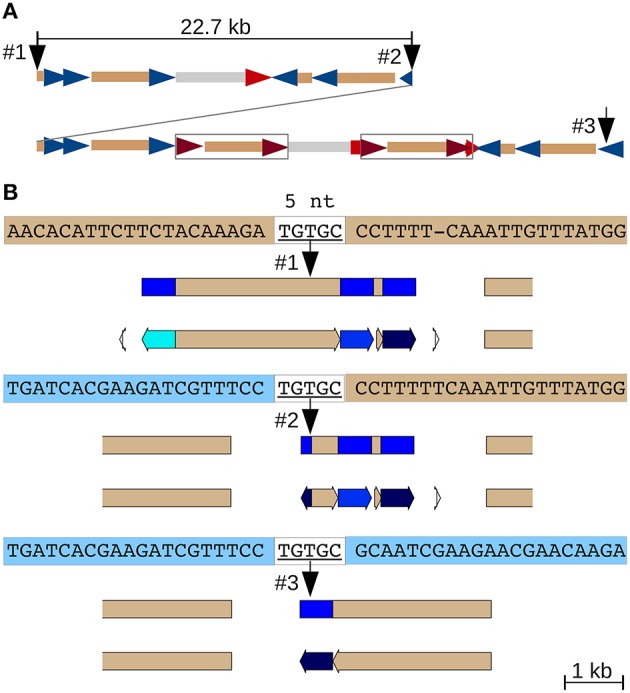
**Alternative end-joining in the formation of an adjacent segmental duplication**. **(A)** Overview of the duplication. A 22.7 kb fragment consisting of several nested and partial CR1s, a CR2 solo LTR and a genomic repeat (gray) was duplicated locally (Figure S3D). Two full-length CR2 elements subsequently inserted into the downstream duplication (boxed). CR1 LTRs blue, CR2 LTRs red and CDS tan. **(B)** A 5 nt match between the polyprotein coding region at the 5′ end of the initial 22.7 kb fragment (top sequence) and the CR1 LTR (represented by the 3′ end of the duplicated region, bottom sequence) likely mediated this tandem duplication by alternative end-joining DNA repair following a double-strand DNA break. JV annotations: CR1 LTRs blue, CDS tan.

A 22.7 kb segmental duplication (Figure 1F) that begins in a CR1 CDS and ends within a CR1 LTR (Figure S3D) features the same five nucleotides at the 5′ end of the upstream and the 3′ end of the downstream duplicated segment, as well as at the junction between them (Figure 4). The most plausible explanation is double-strand DNA breakage followed by single-strand alternative end-rejoining repair (McVey and Lee, 2008) at what is now the duplication junction (between identical 5 nt of the CDS and LTR sequences).

#### A recently formed CentC HOR is the result of an adjacent duplication

**Figure 5:**
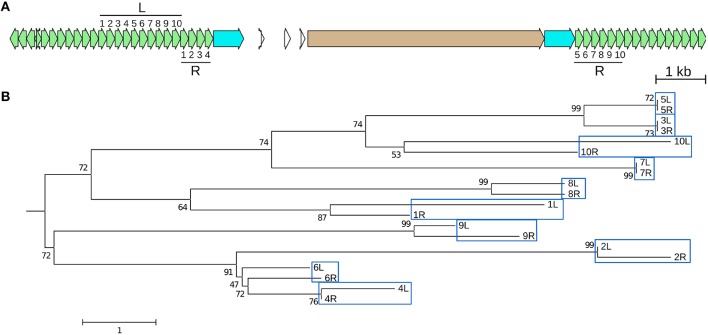
**A recently created CentC HOR is an adjacent duplication**. The youngest (98 kya) of four dated CentC HORs in CEN10 (circled in Figure 1) is a 10-monomer adjacent segmental duplication. **(A)** JV annotation of the duplicated left (L) and right (R) HOR segments with monomers numbered in order. Monomer coordinates are given in Table S6 (2A and 2B). The R HOR is disrupted by a CR1 with identical LTRs (estimated insertion time 0–25.5 kya). **(B)** The L and R monomers are shown in a neighbor-joining tree to indicate that most similar monomers are at equivalent positions in L and R. Bootstrap (1,000) percentages at branch points are shown. The scale represents nucleotide differences. Only monomer pairs 5 and 7 are identical–monomers 3L and 3R differ by one indel. JV annotations: CentC green, CR1 LTR blue, CDS tan.

A phylogenetic tree was constructed (Figure S5) from all full-length CentC monomers (Table S6) of arrays #1 and #3 (Figure 1E) as well as the high quality sequence of array #2 (up to position 1,564,121 nt) to identify HORs. Duplication dates for the longest HORs within each of the three arrays were calculated (Figure 1F) excluding the first and last monomers of each HOR. In addition, all four HORs of a region of array #2 that was resequenced with Sanger technology (see Loss of a CentC Monomer by Homology-Mediated Recombination) in inbreds B73 and Mo17 were dated (boxed in Figure 1F). The seven duplication dates range from 98 to 498 kya. The youngest date is obtained for an adjacent segmental duplication (Figure 5).

#### Loss of a CentC monomer by homology-mediated recombination

**Figure 6:**
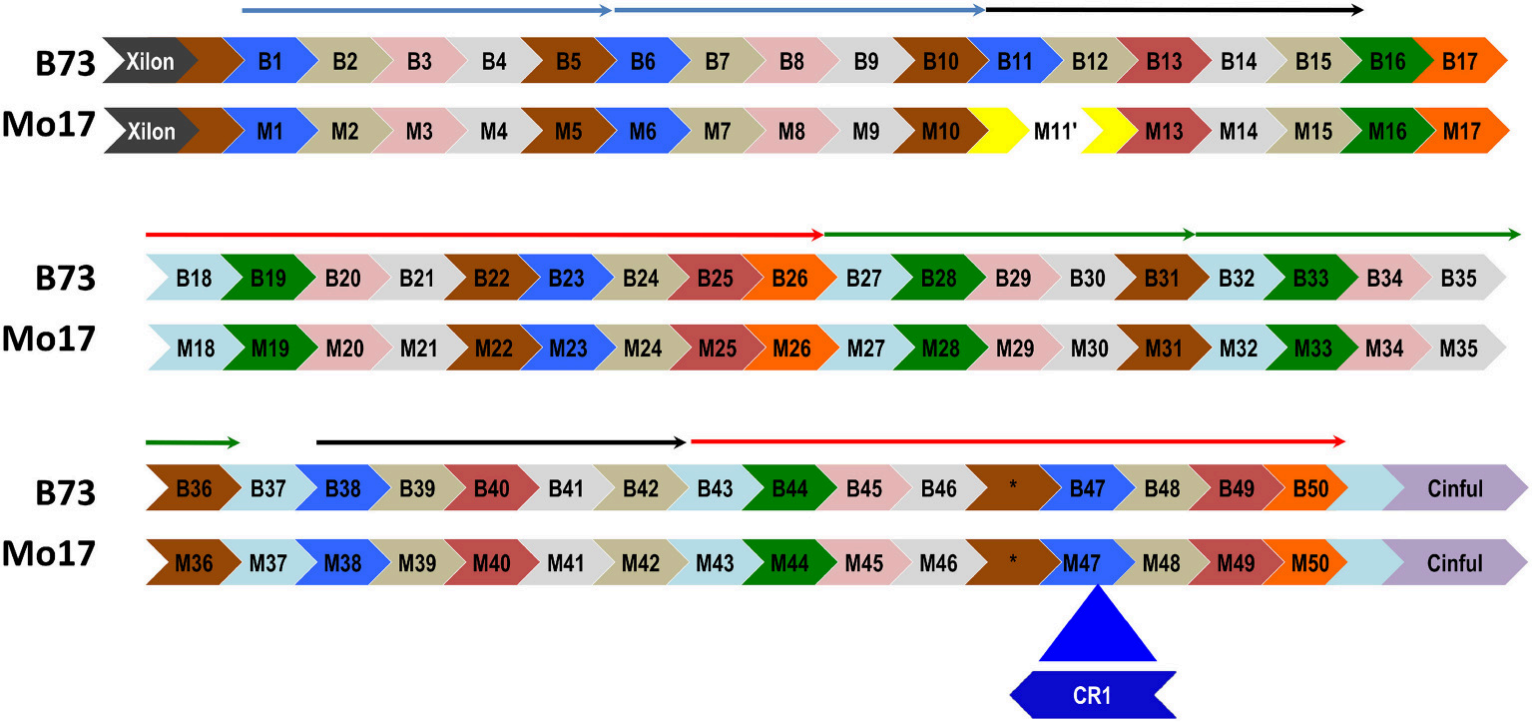
**A CentC monomer is deleted from the array of inbred Mo17**. A distinctive CEN10 CentC array (flanked by Xilon and Cinful retrotransposon insertions) of inbred B73 is aligned with its Mo17 counterpart. Monomers (labeled arrows) are grouped into HORs (thin arrows above monomers) using a phylogenetic tree (Figure S8). Part of the first black HOR is missing in Mo17, where a chimeric monomer (M11′) resulted from a deletion of the 3′ M11 and 5′ M12 monomers. This deletion was mediated by a region of 28 identical nucleotides present in B73 monomers B11 and B12 (Figure S7). The Mo17 CentC array includes a CR1 insertion into M47 that disrupts the second red HOR and is absent in the B73 array. The Mo17 segment downstream of the CR1 insertion to the Cinful element was confirmed by PCR amplification and sequencing. One CentC sequence (^*^) similar to the other brown monomers contains a 40-nt insertion in both inbreds. The youngest (blue) and oldest (green) HORs date to 131 kya and 297 kya, respectively. Potentially recombinant monomers at the ends of HORs were excluded from the alignment used to date the duplication events.

A CentC cluster on BAC #13 consisting of approximately 50 monomers and flanked by Xilon and Cinful retrotransposons, was sequenced to completion using either Sanger or PacBio technology. The two consensus sequences obtained were 100% identical. This CentC array contains 4 HORs that duplicated between 131 and 297 kya (Figure 1F). The youngest and oldest of the four HORs are adjacent segmental duplications (Figure 6 and Figure S6). The corresponding region was cloned and sequenced from maize inbred Mo17 (Data sheet S4) using methods optimized for stabilizing tandem repeats. Comparison of the Mo17 with the B73 sequence revealed the deletion of one CentC monomer from the Mo17 sequence (Figure 6) via a recombination between the monomers M11 and M12 (Figure S7) that resulted in recombinant monomer M11′ formed from the 5′ region of M11 and the 3′ end of M12. This novel chimeric monomer forms its own clade relative to other monomers in the array (Figure S8) and may be unique in the maize genome. A divergence date of 67.9 kya is calculated between the Mo17 and B73 CentCs.

#### Chromosomal inversions have reshaped CEN10

**Figure 7:**
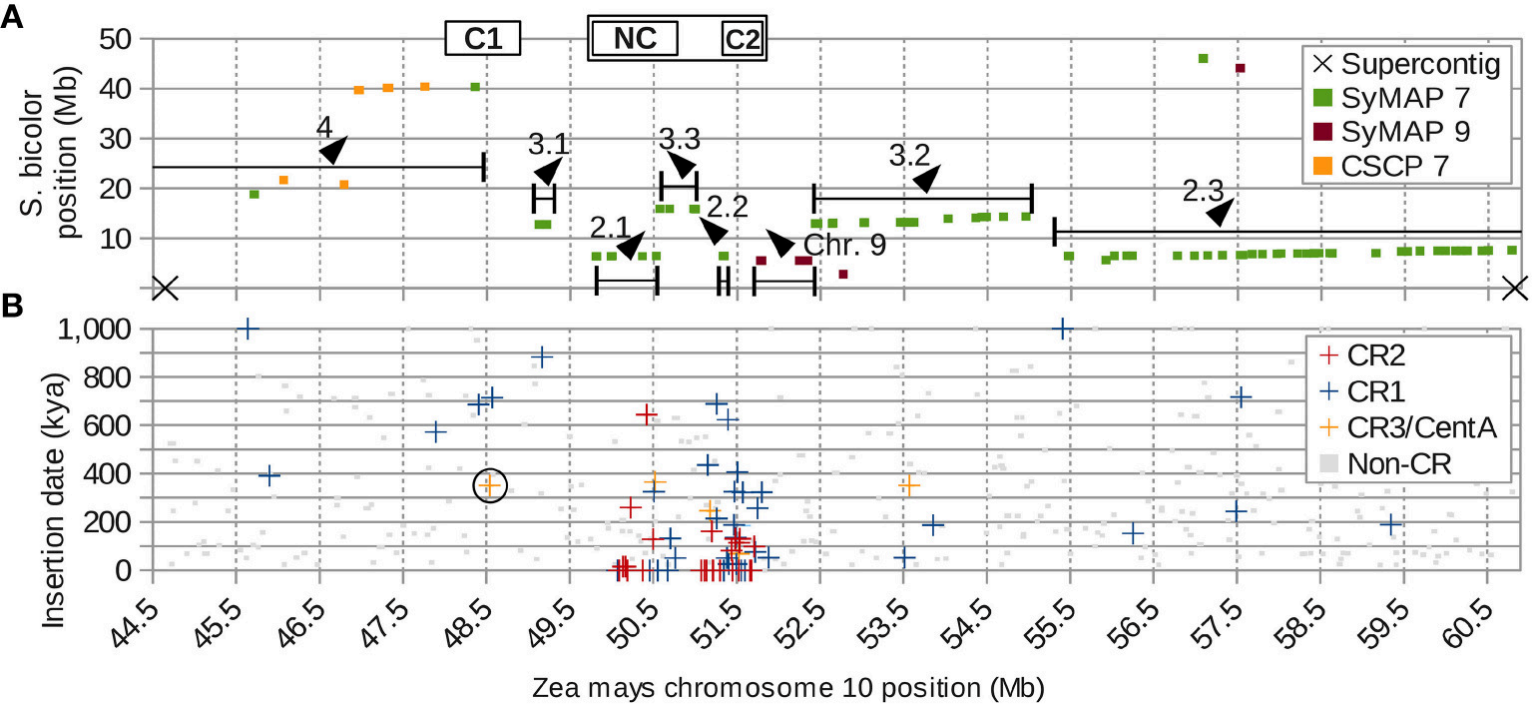
**Supercontig of CEN10 and hemicentric inversions. (A)** Seven jumbled blocks of syntenic sorghum chromosome 7 markers (SyMAP 7) are numbered according to their positions in sorghum, with direction indicated by arrows. Boxed C1 and C2 indicate positions of the CentC clusters, NC indicates the CR cluster with no CentC (Figure 1). The cenH3 binding region CEN10 is marked by the box surrounding NC and C2. Single-copy pericentromere markers conserved between sorghum and maize (CSCP) are shown. CSCP 7 markers of sorghum chromosome 7 are near C1. Sorghum chromosome 9 markers (SyMAP 9) that originated from the end of a larger syntenic block 40 Mb away (Figure S10) were deposited near CEN10 of maize by several inversions involving centromere-proximal DSBs, which are confirmed by the presence of a CR1 element at the distant end of the SyMAP 9 block (Figure S9B). Ends of blocks do not coincide with either of the two supercontig breaks in this region of the reference genome, indicating that breaks in synteny are real and not artifacts of sequence assembly. **(B)** CR and non-CR elements graphed by estimated insertion time and chromosome location indicate that C1 has become inactive >300 kya. The CR element used to date the split of C1 from C2 resulting in inactivation of C1 is circled. CR3/CentA = CR3 and CentA (nonautonomous CR3).

The inversion that split the original CentC cluster into two regions resulted in one inactive (C1) and one active (C2) CentC cluster. CR elements continued to insert only into the active CentC cluster, thus the time of inversion can be dated to ~350 kya based on the most recent CR insertion in C1. Additional inversions, including some very recent ones (< 16.4 kya), moved CR sequences to the upstream border of the current centromere and a previously pericentric region to the CEN10 segment between NC and C2 (Figure 7 and Figure S9B).

In addition to the hemicentric inversion that split the original CentC cluster into C1 and C2, a number of other inversions can be reconstructed based on sorghum microsynteny. The CEN10 reassembly contains synteny markers that are up to 9 Mb apart and discontiguous in sorghum chromosome 7 (Figure 1H). These markers are members of larger syntenic blocks spanning ~45 Mb in maize chromosome 10 (Figure S10). CEN10 includes the ends of sorghum chromosome 7 syntenic blocks 2 and 3 (of 4) and markers from the end of a syntenic sorghum chromosome 9 block that is ~40 Mb downstream. These remnants of larger syntenic blocks were moved into CEN10 by multiple inversions. Syntenic blocks 2 and 3 are heavily scrambled (subparts 3.1, 2.1, 3.3, 2.2, 3.2 and 2.3 in Figure 7) relative to their positions in sorghum, indicating that several additional inversions occurred during maize CEN10 evolution. In total, at least 8 and possibly 17 inversions (assuming CR insertions follow the active centromere) are needed to explain the current arrangement of sorghum syntenic markers in and around CEN10 (Figure S9).

## Discussion

The high quality sequence of a complete maize centromere represents a significant advance over the current, highly fragmented reference genome consisting of unordered and unoriented sequence contigs, and allows a detailed study of centromeric repeats and genomic rearrangements in the centromere. Retrotransposon-spanning PacBio reads provide certainty about both TSD sequences for each CR element, which is helpful in untangling the complicated rearrangements we document for the elements in CEN10 and in resolving the large tandem duplications. Moreover, this new sequence provides certainty about order and orientation of syntenic markers and thus reconstruction of recent genomic rearrangements. Whole-genome shotgun sequencing with longer reads of higher quality, combined with other novel physical mapping technologies, may enable closure of the remaining gaps in the future.

Complete centromere sequence of similar length and quality is only available for the rice CEN8 (Nagaki et al., 2004; Wu et al., 2004, 2009), which is also characterized by inverted tandem repeat arrays, numerous CR elements and the presence of active genes. Furthermore, segmental duplications and numerous centromere-proximal inversions distinguish CEN8 of different rice species or subspecies (Ma and Bennetzen, 2006; Ma and Jackson, 2006; Ma et al., 2007), indicating that these events are not specific to the recently polyploidized maize genome with its large chromosomes.

### Efficient DSB repair is a major force in centromere repeat evolution

Comparison of a small CentC array in CEN10 that is flanked by a Xilon and a Cinful retrotransposon and was sequenced to high quality in both B73 and Mo17 inbreds revealed the insertion of a CR1 element into, and removal of a single CentC monomer from, the CentC array of Mo17. Deletion of that CentC monomer almost certainly occurred via homology-based intrastrand DSB repair rather than gene conversion, as the latter would have required nearly perfect (off-by-one-repeat) pairing of the CentC monomers between sister chromatids or orthologous chromosomes. If such a pairing mechanism exists in centromeres, it would be surprising if it did not require the lining up of the upstream xilon element (< 2 kb away) to restore the precise CentC order. Also, the fact that the break points of 14 rice centromere misdivision events mapped to the middle of CentO arrays (Cheng et al., 2002) supports the frequent formation of DSB in centromeres.

The instantaneous formation of a novel CentC monomer by recombination provides an important mechanism for the rapid evolution of tandem centromere repeats. It also suggests that the prevalence of tandem DNA repeats at the centromeres of many eukaryotic chromosomes is a result of selection for a substrate that allows efficient repair of frequent DSB in and near centromeres caused by mechanical shear exerted on the DNA in the proximity of the spindle microtubules (Guerrero et al., 2010). DSB repair was proposed as the mechanism that generated a number of novel CR1 recombinants in maize (Sharma et al., 2008) and a series of novel tandem repeats near CEN9 that were derived from CR1 (Sharma et al., 2013), but the extent of DSB repair that occurs at centromeres has only become apparent with the high quality sequence available now.

Divergence of the B73 from the Mo17 CEN10 based on this CentC region is estimated at 67.9 kya, which is substantially higher than the previous estimate of 10.3 kya obtained from HapMap2 (Chia et al., 2012) data of a non-recombinant flanking region (Schneider et al., 2016), raising the possibility that imprecise homology-mediated repair of CentC islands may result in accelerated mutation rates in tandem repeats. However, although unlikely, we cannot entirely exclude the possibility that the B73 and Mo17 CentC clusters are paralogs rather than orthologs.

Alternative end-joining accounts for many of the CEN10 features including (1) solo CR LTRs, (2) internal CR deletion (Figure 2) and CentC monomer deletion (Figure 6), (3) added CR coding sequence (Figure 3) and the 22.7 kb segmental duplication (Figure 4), and (4) adjacent duplications resulting in CentC HORs (Figures 5, 6). An alternative end-joining model has been proposed using microhomology-mediated end joining (MMEJ) as a mechanism (McVey and Lee, 2008). Polymerase Θ, an error prone polymerase (Arana et al., 2008) has been specifically implicated in the mechanism of MMEJ (Kent et al., 2015). This polymerase suppresses crossover recombination and causes the cell cycle to be stalled when silenced (Ceccaldi et al., 2015). It is conceivable that special DNA repair mechanisms have evolved for the centromere regions of plants that require frequent repair.

### Hemicentric inversions shrink centromeres and place genes into their immediate vicinity

We document a number of hemicentric inversions, i.e., involving one break in the active centromere and a second on the chromosome arm, in CEN10. Inversions like the one that split the initial CentC cluster into C1 and C2 (Figures 1, 7) have the potential to dramatically reduce the size of the active centromere and require expansion of the cenH3 nucleosomes to regions flanking the CentC cluster to restore centromere size. This appears to be what has happened at the NC region of CEN10, which is characterized by a large number of recently inserted CR elements. Hemicentric inversions of relatively short fragments shuffle pericentromeric regions, likely with relatively limited effect, but those involving long regions (e.g., the SyMAP 9 markers from ~40 Mb away) can place important genes into, or immediately next to, the active centromere (gene 3), and create the potential for strong selection for specific centromeres due to the linked gene. Furthermore, actively transcribed genes bind lower amounts of cenH3 (Yan et al., 2006), and may restrict centromere expansion in that direction (Wang et al., 2014). The actively transcribed gene 3, which is >20 kb in length, may be responsible for limiting cenH3 expansion at the downstream border of CEN10.

Hemicentric inversions have been reported in maize, in one case (discovered by FISH) involving 20% of the long arm of chromosome 8 that still resulted in fertile heterozygotes (Lamb et al., 2007b) and in other cases discovered by rearranged pericentromeric markers (Wang and Bennetzen, 2012). Our careful analysis of maize CEN10 reveals hemicentric inversions to be quite frequent in maize. Using the manually derived series of proposed inversions (Figure S9B), at least 9 CEN10-proximal inversions need to be invoked since the CentC split to account for all breakdowns of sorghum-maize synteny, yielding a rate of 1 inversion per < 38.9 kya.

### Different evolutionary forces in centromeres

Our results indicate that, in centromeres, sequence evolution by DSB-induced rearrangement (deletions, duplications, inversions, insertion of non-syntenic genes, or organellar DNA, and the creation of recombinant retrotransposons and tandem centromere repeat variants) outpaces that by single nucleotide mutations. For these and other reasons (e.g., Muller's ratchet (Bowers et al., 2005)) centromeres are bad neighborhoods for genes. Conversely, genes are bad for centromeres, as they disrupt the periodicity of tandem repeats and reduce cenH3 binding if transcribed. Thus, the division of chromosomes into distinct gene-poor heterochromatic pericentric, and gene-rich euchromatic, regions is a logical consequence of these mutually antagonistic effects. Hemicentric inversions have the potential to disrupt this chromosomal organization of distinct territories. Similarly, deletion of existing centromere sequence followed by cenH3 relocation can place genes and centromeres in close proximity (Schneider et al., 2016). Our ability to measure how genes and centromeres impact each other and possibly affect speciation will improve as additional complete centromere sequences are obtained for other chromosomes and inbreds.

## Author contributions

TW, GP, and KS wrote and edited the manuscript. TW and GP assembled, analyzed and annotated the CEN10 sequence and generated figures and tables. MN and GP sequenced and assembled Mo17 sequence. AS identified the missing BAC. AS and RX isolated BAC DNA for sequencing. ZX isolated centromeric DNA via chromatin immunoprecipitation. PA and JB performed FISH. RKD suggested, and PB and JR contributed to, initial attempts to correct PacBio reads with Illumina data.

### Conflict of interest statement

The authors declare that the research was conducted in the absence of any commercial or financial relationships that could be construed as a potential conflict of interest. The reviewer AH and the handling Editor declared a shared affiliation, and the handling Editor states that the process nevertheless met the standards of a fair and objective review.
