# Supplementary material for: High Quality Maize Centromere 10 Sequence Reveals Evidence of Frequent Recombination Events
Source: Front Plant Sci. 2016 Mar 23;7:308. doi: 10.3389/fpls.2016.00308 (PMC4806543; doi:10.3389/fpls.2016.00308)
Supplement: Supplementary file 12 [file Image2.PDF]

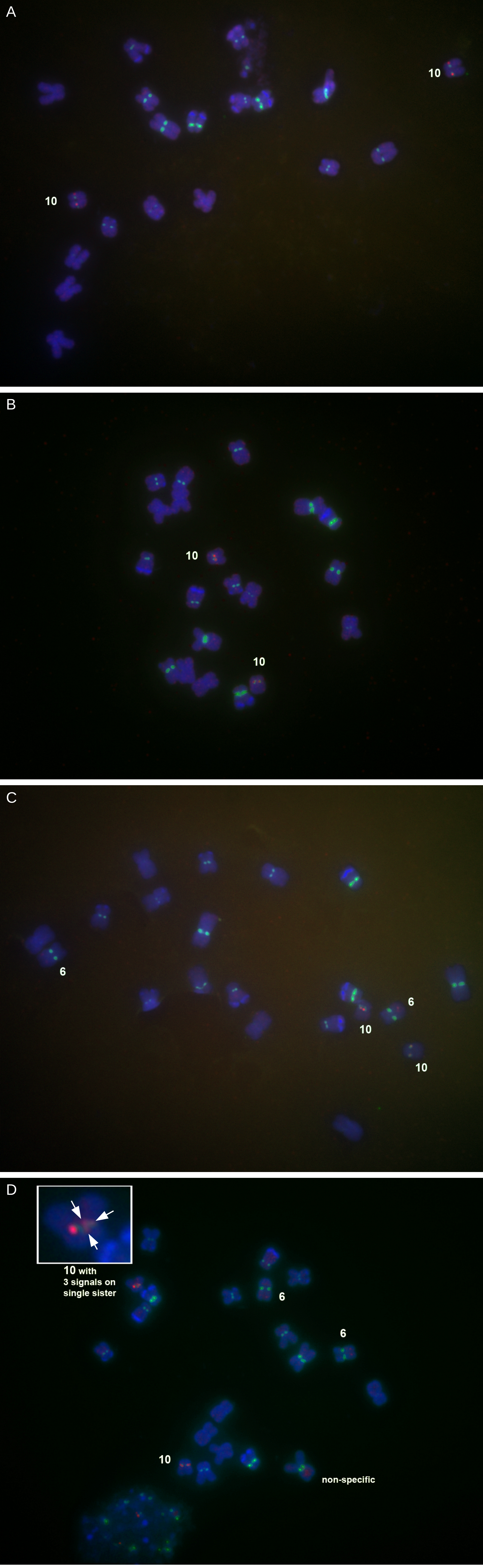

Figure S2. FISH confirms the presence of genes in the centromere. CEN10 probes generated from positions 991,393-999,754 nt, 1,331,762-1,340,808 nt, and 1,771,713-1,780,104 nt all localize near CentC (green) and correspond to genes 1, 2, and 3 (Figure 1), respectively. In agreement with our assembly, individual gene probes show that gene 1 (A) is farther from CentC than gene 2 (B) and gene 3 (C). (D) A mixture of all three gene probes. Part (D) includes an enlargement (boxed) showing all three gene signals (arrows) on one sister chromatid. Genic probes are labeled red, CentC in green and DAPI stained chromosomes are blue. Some non-specific hybridization of the gene 3 probe to the arm of chromosome 6 is consistent with previously reported synteny between sorghum chromosome 9 in both maize chromosomes 10 and 6 (Wang and Bennetzen 2012).
