# Supplementary material for: High Quality Maize Centromere 10 Sequence Reveals Evidence of Frequent Recombination Events
Source: Front Plant Sci. 2016 Mar 23;7:308. doi: 10.3389/fpls.2016.00308 (PMC4806543; doi:10.3389/fpls.2016.00308)
Supplement: Supplementary file 13 [file Image3.PDF]

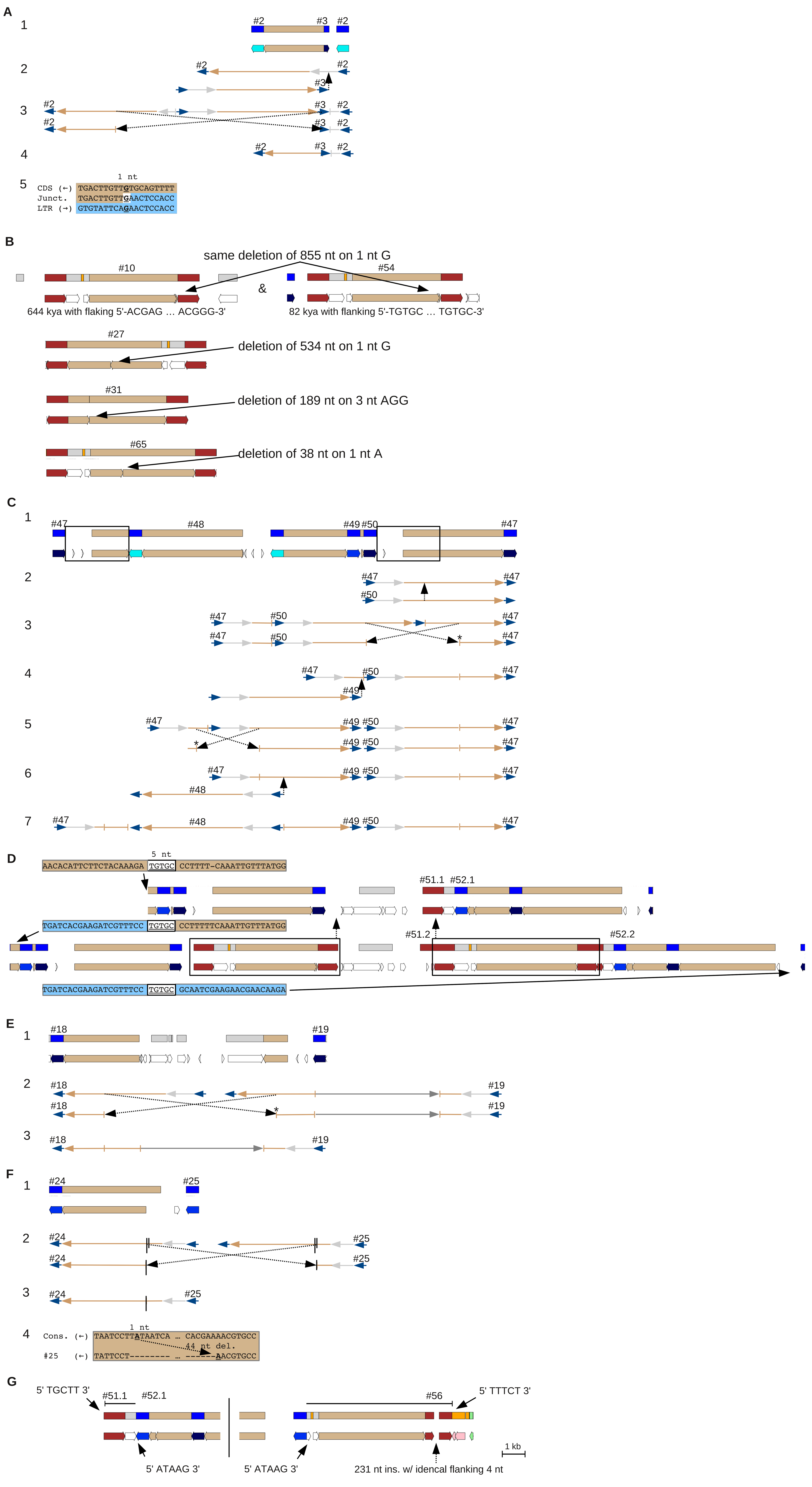

Figure S3. Additional CRs showing local deletions, duplications, or chimerism. (A) Deletion resulting from recombination with a nested element. CR element #3 (Table S5) inserted into the UTR of CR #2, then a recombination between the 3' LTR of CR #2 and the CDS of CR #3 led to a deletion. The junction at the apparent CDS/LTR recombination point has a single nucleotide overlap between CR1 consensus sequences. (B) Five other internal deletions discovered in CR2 elements of CEN10. CR #10 and #54 have the same 855 nt deletion but are located 1 Mb from each other and inserted 562 kya apart (Table S5). (C) Apparent duplication of coding sequence (boxed) resulting from two recombinations. CRs #50 and #49 inserted into #47, then recombined between their CDSs leading to the loss of one LTR for CRs #50 and #49 as well as the apparent duplication of CDS in CR #47. (D) Annotations describing the complete left (top) and right (bottom) 22.7 kb duplicated segments of CEN10. Two CR2s inserted into the right part, which is illustrated by boxing the inserted elements and indicating their original insertion positions relative to the left segment. Sequences at the borders of the duplicated segments are shown. (E)-(G) Chimeric CRs resulting from recombination between two different CRs. For (G) mismatched TSDs are indicated outside the CR2 LTRs (#51.1 and #56). The same UTR sequence (TSD) flanks a nested CR1 (#52.1) in the chimeric CR indicating contiguity of the chimeric element. However, there is a 231 nt insertion in its 3' LTR (#56). Numbers above CR annotations are their identifiers in Table S5. For (A), (C), (E), and (F) the JV image is part 1 followed by a series of parts with line drawings describing events leading to their current state in CEN10 (* = insertion point arbitrarily estimated). JV annotations are described in Figure 2. Additionally for (A) and (F) their last parts show recombination junction points relative to consensus sequences derived from all uninterrupted CRs in CEN10.
