# Supplementary material for: High Quality Maize Centromere 10 Sequence Reveals Evidence of Frequent Recombination Events
Source: Front Plant Sci. 2016 Mar 23;7:308. doi: 10.3389/fpls.2016.00308 (PMC4806543; doi:10.3389/fpls.2016.00308)
Supplement: Supplementary file 14 [file Image4.PDF]

A

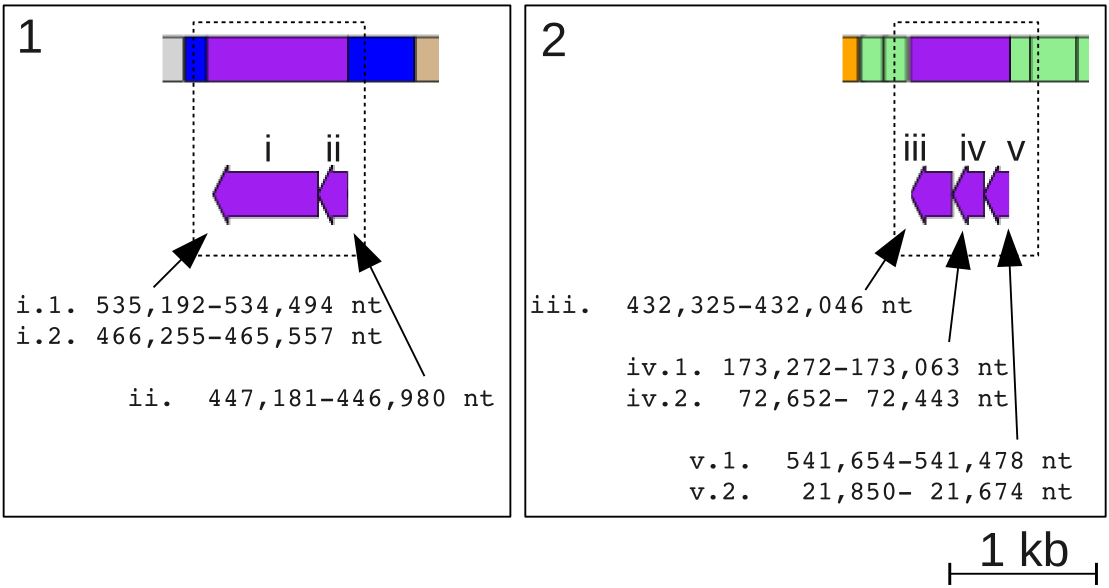

B

Junction of i & ii: TAAAGT AG GGAA GAAAGA
Mito. seq. in i: TAAAGT *GT* GGAA ATAGTC
Mito. seq. in ii: GCTCCG AG GGAA GAAAGA

Junction of iii & iv: TGCATG G C TCTCGG
Mito. seq. in iii: TGCATG G *T* CGTCAC
Mito. seq. in iv: TGGCCT C C TCTCGG

Junction of iv & v: TGCGCC AGT CCGCAA
Mito. seq. in iv: TGCGCC AGT AAGTAG
Mito. seq. In v: CGTCGT AGT CCGCAA

Figure S4. Mitochondrial sequences in CEN10 may have recombined. Two ~1 kb segments of mitochondrial DNA that inserted into CEN10 (see Figure 1E) from five different regions of the 570 kb mitochondrial genome. (A) JV images including two segments (purple boxes in parts 1 and 2) that consist of sequences homologous to regions in the mitochondrial genome (arrows i-v). Sequences i, iv, and v each align with two (duplicated) regions in the mitochondrial genome. CEN10 nucleotide positions are i: 1,360,840-1,361,538 nt, ii: 1,361,533-1,361,734 nt, iii: 1,477,247-1,477,526 nt, iv: 1,477,527-1,477,736 nt, and v: 1,477,734-1,477,910 nt. (B) Sequences at junctions between i-v are aligned to reference mitochondrial sequences. Underlined nucleotides are matching between the CEN10 nuclear sequence and parts of the mitochondrial reference genome, suggesting homology-mediated recombination at least between iv and v. Italicized nucleotides indicate differences at potential overlaps for the i & ii and iii & iv junctions. JV annotations are described in the methods – here the purple mitochondrial sequences are flanked either by blue CR1 LTR or green CentC.
