# Supplementary material for: High Quality Maize Centromere 10 Sequence Reveals Evidence of Frequent Recombination Events
Source: Front Plant Sci. 2016 Mar 23;7:308. doi: 10.3389/fpls.2016.00308 (PMC4806543; doi:10.3389/fpls.2016.00308)
Supplement: Supplementary file 15 [file Image5.PDF]

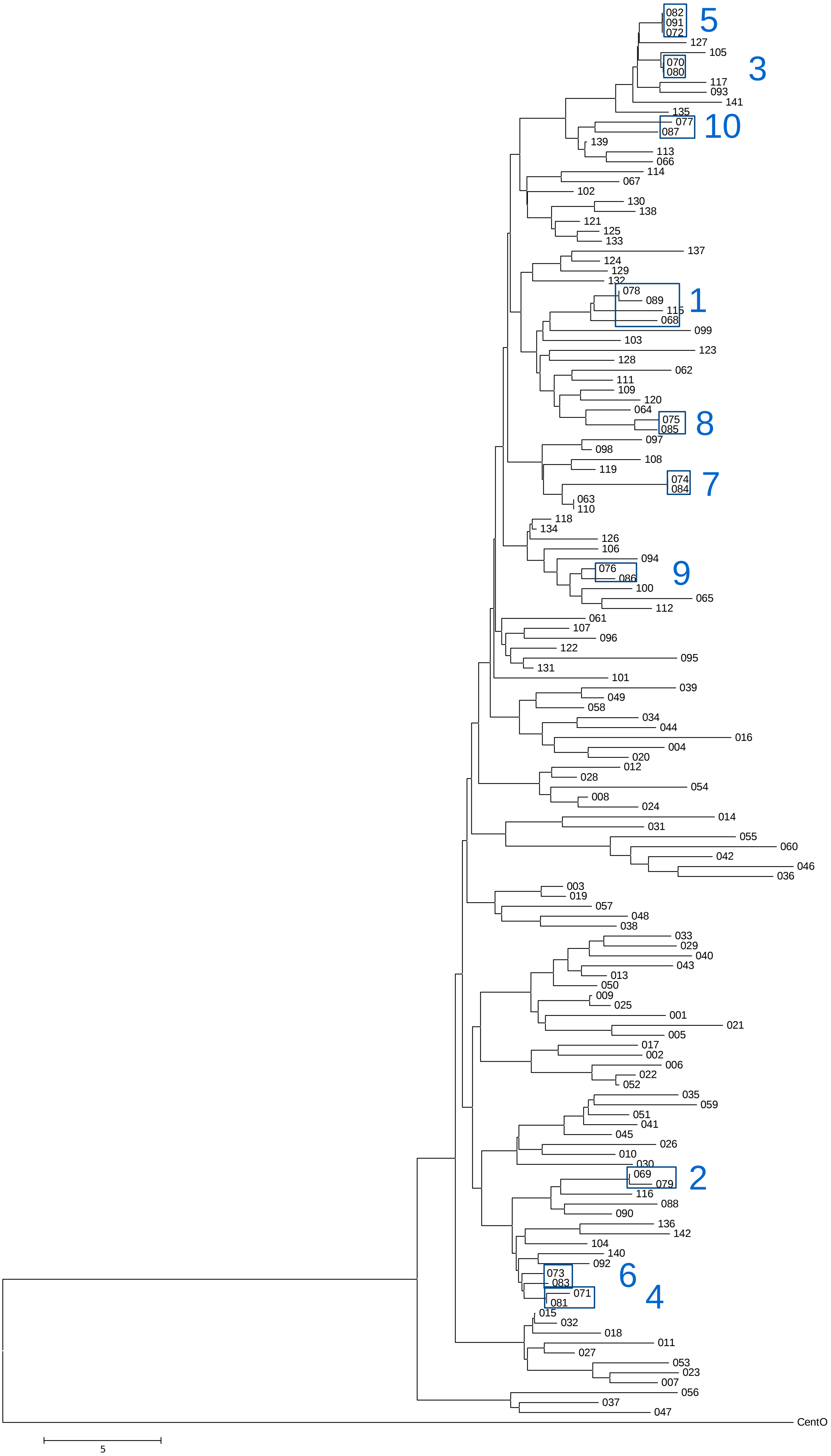

Figure S5. Full-length CentC monomers can be grouped into HORs. This neighbor-joining phylogenetic tree of 142 full-length CentC monomers (numbered according to their positions in CEN10) is used to identify HORs. Monomers from Figure 5 (HOR “2” in Table S6) are boxed and labeled (blue numbers) – the HOR starts with pair #68/#78 and continues to pair #77/#87. Tree bootstrap is 1,000 and scale indicates nucleotide differences.
