# Supplementary material for: High Quality Maize Centromere 10 Sequence Reveals Evidence of Frequent Recombination Events
Source: Front Plant Sci. 2016 Mar 23;7:308. doi: 10.3389/fpls.2016.00308 (PMC4806543; doi:10.3389/fpls.2016.00308)
Supplement: Supplementary file 16 [file Image6.pdf]

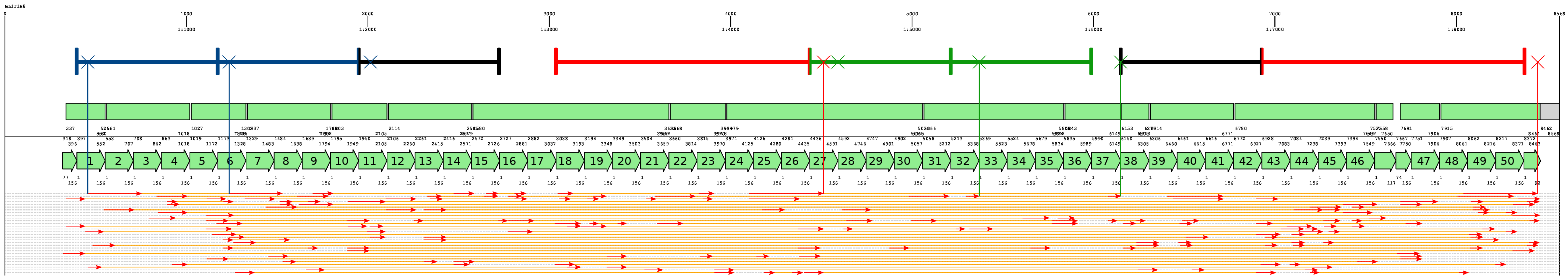

Figure S6. JV image of the B73 CentC array. CentC HORs as defined by full-length CentC monomers are marked above the JV annotations by blue, black, red, and green colored lines as in Figure 6. Alternatively, HOR borders can be defined using the longest MUMmer annotations on the edges of monomer-defined HORs (Xs). The youngest (blue) and oldest (green) HORs date to 131 and 297 kya, respectively. CentC monomers are indicated by green arrows and numbered consecutively as in Figure 6 and Figure S8. The longest perfect matches identified by MUMmer are indicated by red arrows below the numbered CentCs.
