# Supplementary material for: High Quality Maize Centromere 10 Sequence Reveals Evidence of Frequent Recombination Events
Source: Front Plant Sci. 2016 Mar 23;7:308. doi: 10.3389/fpls.2016.00308 (PMC4806543; doi:10.3389/fpls.2016.00308)
Supplement: Supplementary file 17 [file Image7.PDF]

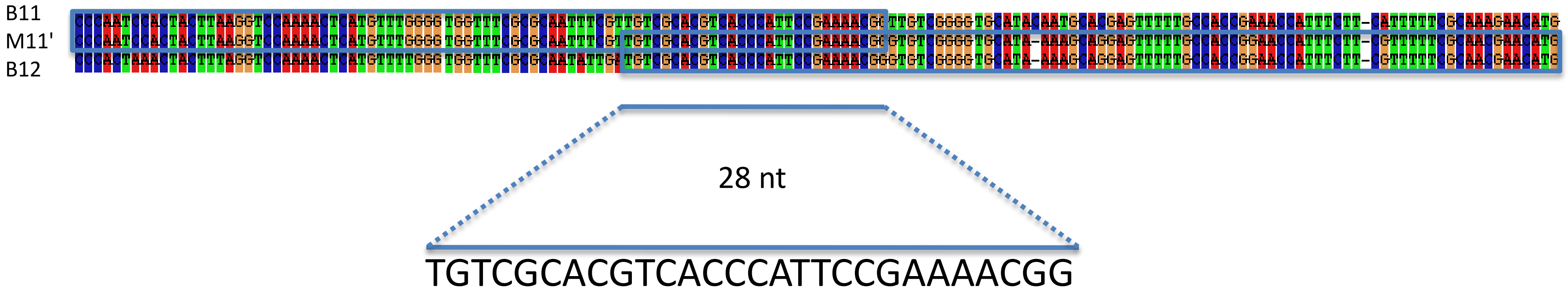

Figure S7. Recombinant CentC monomer in inbred Mo17. Deletion of parts of the M11 and M12 monomers formed chimeric M11' in maize inbred Mo17, a completely new CentC variant that consists of the 5' end of M11 and the 3' end of M12. The multiple sequence alignment of monomers B11, M11', and B12 defines the coordinates of the recombination event. A 28 nt region of sequence identity is shared where recombination between ancestral M11 and M12 led to the formation of M11'.
