# Supplementary material for: High Quality Maize Centromere 10 Sequence Reveals Evidence of Frequent Recombination Events
Source: Front Plant Sci. 2016 Mar 23;7:308. doi: 10.3389/fpls.2016.00308 (PMC4806543; doi:10.3389/fpls.2016.00308)
Supplement: Supplementary file 18 [file Image8.PDF]

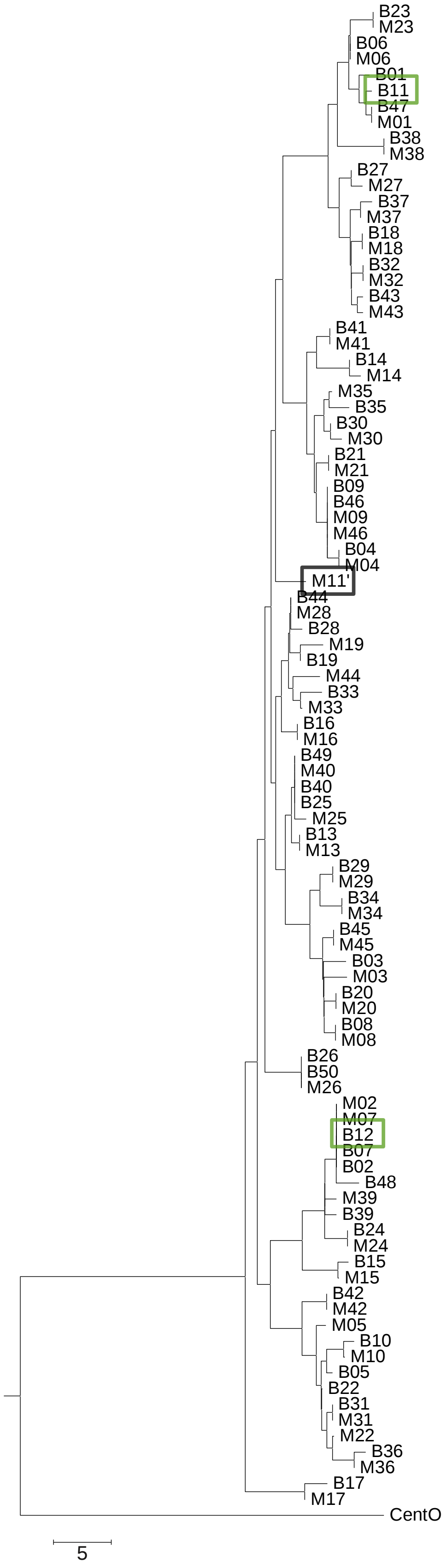

Figure S8. Phylogenetic tree for comparing monomers of a CentC island sequenced from two inbreds. The 1st through the 10th monomers of equivalent CentC arrays in B73 and Mo17 match, after which there is mismatch due to the loss of a single CentC monomer in Mo17. This loss creates chimeric monomer M11' (boxed black) homologous to the first half of B11 and second half of B12 (boxed green). Monomers then match again until the last Mo17 monomer before a CR insertion (M45 with B45). The neighbor-joining tree was bootstrapped 1,000 times and the scale represents nucleotide differences.
