# Supplementary material for: High Quality Maize Centromere 10 Sequence Reveals Evidence of Frequent Recombination Events
Source: Front Plant Sci. 2016 Mar 23;7:308. doi: 10.3389/fpls.2016.00308 (PMC4806543; doi:10.3389/fpls.2016.00308)
Supplement: Supplementary file 19 [file Image9.PDF]

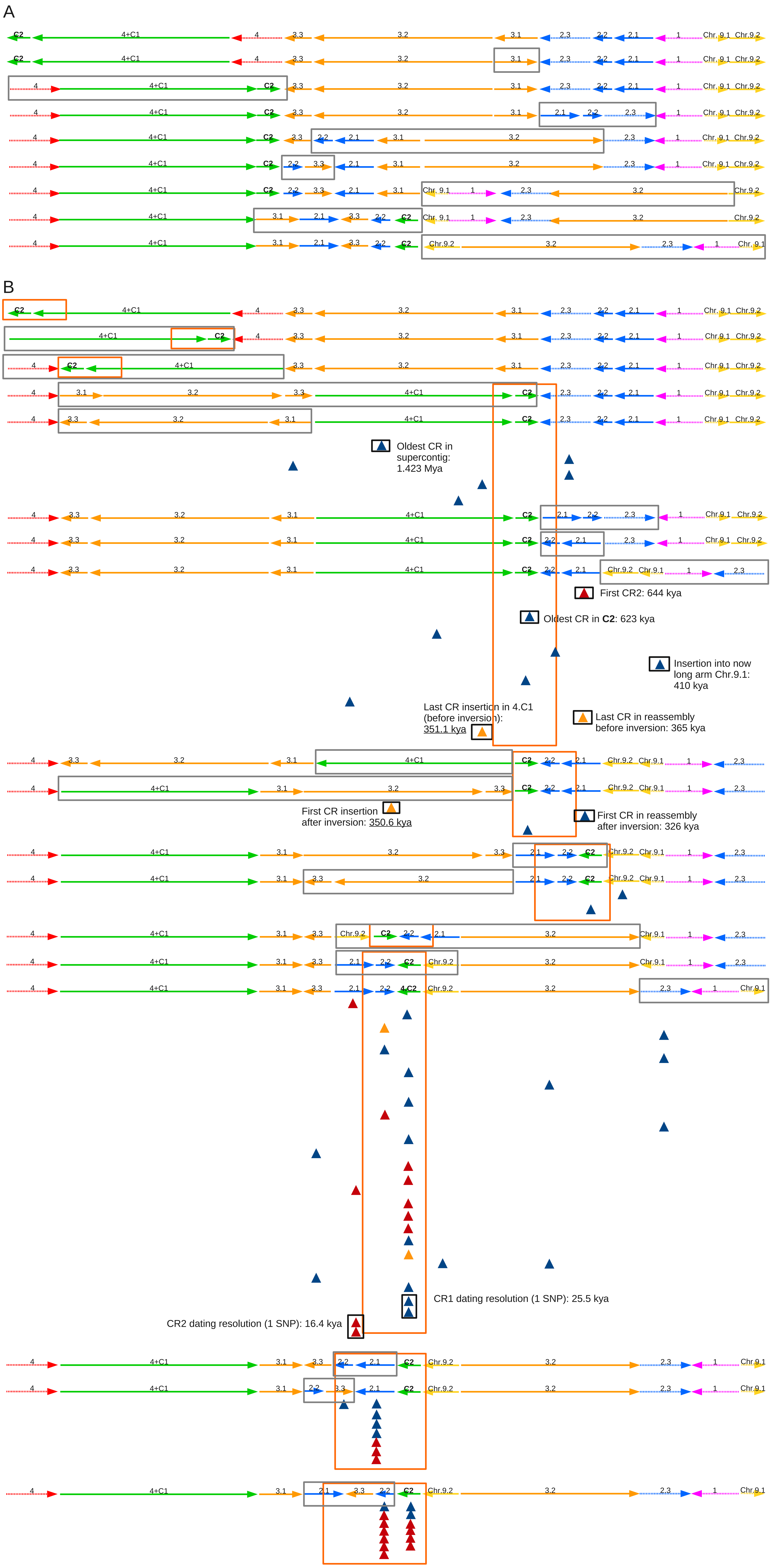

Figure S9. Numerous inversions can explain the arrangment of centromere 10 syntenic blocks. (A) A minimal series of 8 inversions can explain the arrangement of sorghum syntenic markers around CEN10 starting from an ancestral state (top row) having syntenic blocks ordered as they are in present-day sorghum. (B) If the positions and dates of CR insertions are considered, a series of 17 inversions can be made by following insertions around the centromere/"C2" (orange boxes). Sorghum chromosome 7 syntenic blocks are shown as arrows with numbers as described in Figure 7 and Figure S10, and the (current) centromere proximal or distal sorghum chromosome 9 blocks labeled Chr.9.2 or Chr.9.1, respectively. Block 4 differs from 4+C1 by being outside the supercontig containing CEN10. Solid arrows are approximately to scale and stippled arrows (blocks 4, 2.3, 1, and Chr.9.1) are reduced to fit the figure. Inversions are boxed in gray. CR insertions (CR1 = blue, CR2 = red, CentA = orange) are illustrated under the syntenic blocks into which they inserted in order (top is oldest), except for the last two inversions where all CRs have identical LTRs. CR elements that inserted between blocks 3.3 and 2.2 were assigned to block 2.2.
