# Supplementary material for: High Quality Maize Centromere 10 Sequence Reveals Evidence of Frequent Recombination Events
Source: Front Plant Sci. 2016 Mar 23;7:308. doi: 10.3389/fpls.2016.00308 (PMC4806543; doi:10.3389/fpls.2016.00308)
Supplement: Supplementary file 20 [file Image10.PDF]

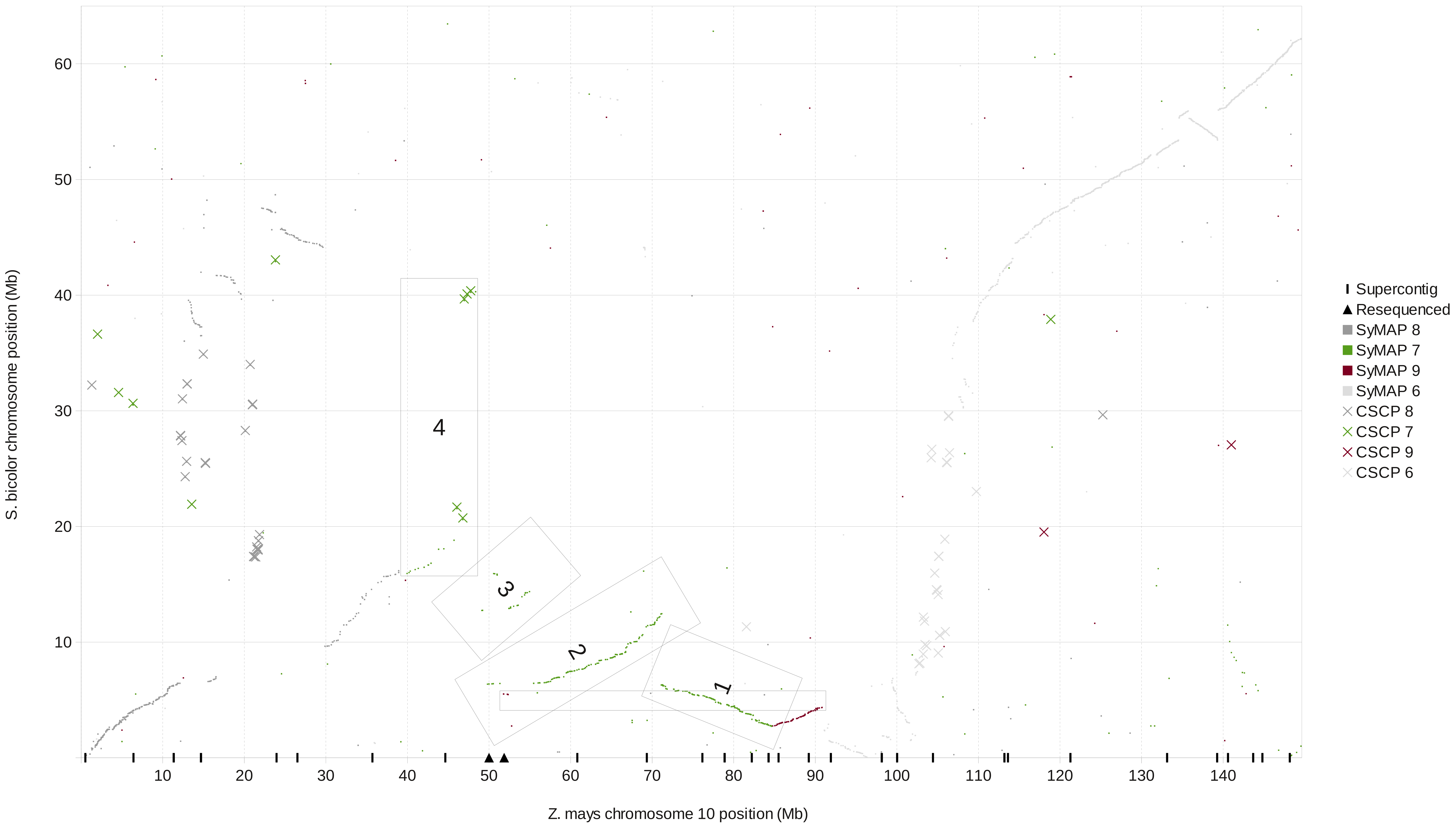

Figure S10. The ends of larger syntenic blocks are in CEN10. Four sorghum chromosome 7 syntenic blocks (numbered boxes) make up the bulk of ~50 Mb in the middle of maize chromosome 10, where there also appears sorghum chromosome 9 syntenic markers. CEN10 (between "Resequenced" points) includes syntenic markers from the ends of sorghum chromosome 7 blocks 2 and 3, as well as one end of the sorghum chromosome 9 block ~40 Mb away (long horizontal box). Sorghum syntenic markers (SyMAP) and sorghum pericentric marker (CSCP) labels are followed by sorghum chromosome number in the legend. Supercontig breaks (1,000 contiguous Ns in the reference genome) are plotted and are typically spanned by the larger syntenic blocks (borders of blocks are not caused by breaks in the physical map).
